# An observational study on the impact of the socio-economic crisis in greece on ICU patient recruitment

**DOI:** 10.1186/2197-425X-3-S1-A137

**Published:** 2015-10-01

**Authors:** S Savvidou, K Marmanidou, M Oikonomou, D Matamis

**Affiliations:** 'Papageorgiou' General Hospital of Thessaloniki, ICU, Thessaloniki, Greece

## Introduction

The recent ongoing financial crisis in Greece has resulted in an overall significant reduction in road traffic accidents, severe injuries and deaths.[1] On the other hand, there is conflicting data about the rate of suicides and suicidal attempts.[2, 3]

## Objectives

To investigate the impact of the Greek socioeconomic crisis that started in 2010 on the ICU and hospital admissions due to traffic accident related multiple trauma (MT) and admissions after suicidal attempt (SA).

## Methods

“Papageorgiou” General Hospital is one of the four major hospitals of Thessaloniki, the second largest Greek city of 1.2 million inhabitants. Data was extracted from hospital databases (ICD-10 diagnoses V01-V89 for MT and X60-X84 for SA) from the year 2008 to 2014. Several variables concerning patient demographics, APACHE and SAPS II scores, length of ICU stay (LOS) and outcome were recorded and further statistically analyzed using χ[2], Mann-Whitney and Kruskal-Wallis tests.

## Results

**Figure 1:**
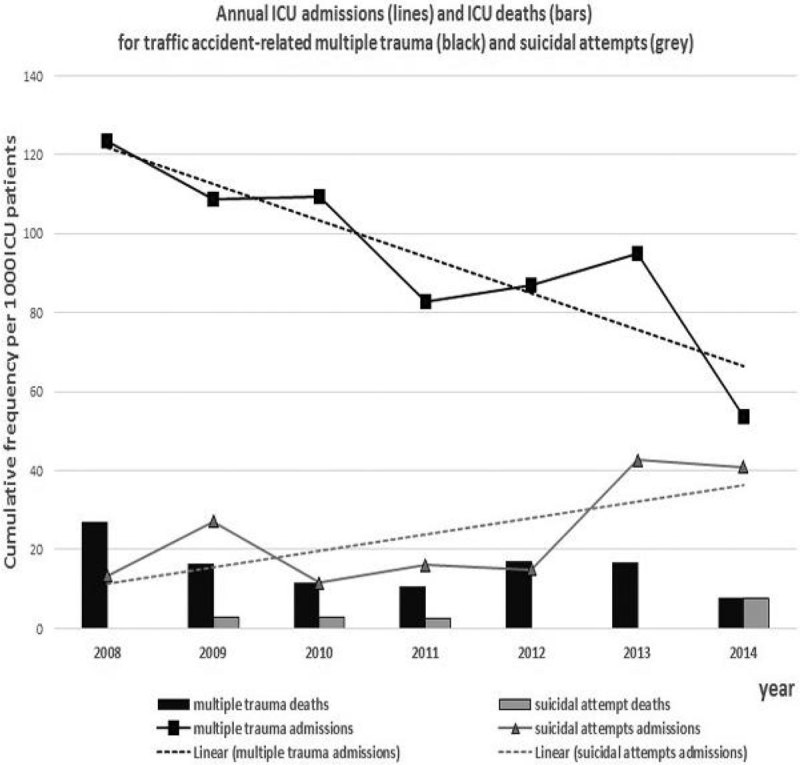
Figure 1

During 2008-2014 the hospital has welcomed an average of 100.2+4.7x10^3^ patients annually, with an increasing trend. The general adult multidisciplinary ICU has 10 beds, 92.4% percentage of mechanically ventilated patients and an average admission rate of 392.4+41.6 patients per year. Over the study period, 262 patients with MT and 66 patients after SA were admitted in ICU. MT patients were 79.8% males, mean age 40+19.3 years old, median LOS 10 days (interquartile range 4-22), mean APACHE score 10.9+6.6, mean SAPS II 37.6+16.7, overall mortality 16.8% with 48h mortality of 7.6%. SA patients were 62.1% females, mean age 43.1+15, median LOS 2 days (interquartile range 1-4), mean APACHE score 13.3+7.3, mean SAPS II 44.7+13.8,overall mortality 9.1% with 48h mortality of 6.1%. There was a significant decrease (-45.5% in total or -7.59% per year) in the admission rate of MT along with a significant increase (+68.9% in total or +11.49% per year) in the admission rate of SA (actual numbers and trend-lines shown in Figure 1). Statistical analysis revealed associations of mortality with age (p = 0.001 for MT and p = 0.016 for SA) and with both scoring systems (p < 0.001). No statistical significant differences of gender, age, LOS and mortality were recorded over time.

## Conclusions

The financial crisis in Greece has resulted in a gradual and ongoing decrease in ICU admissions of patients with multiple trauma after severe traffic accident and a parallel increase in admissions after suicidal attempt. Overall mortality has been kept constant over time.
